# Development of a Blue Emitting Calcium-Aluminate Phosphor

**DOI:** 10.1371/journal.pone.0162920

**Published:** 2016-09-20

**Authors:** Doory Kim, Han-Eol Kim, Chang-Hong Kim

**Affiliations:** 1 Department of Chemistry, University of California, Berkeley, California, United States of America; 2 Gwangju Institute of Science and Technology, Gwangju, South Korea; 3 Korea Institute of Science and Technology, Seoul, South Korea; Institute of Materials Science, GERMANY

## Abstract

We report methodological advances that enhance the phosphorescence efficiency of a blue-emitting calcium aluminate phosphor (CaAl_2_O_4_: Eu^2+^, Nd^3+^). The investigation of long-persistence blue-emitting phosphors is highly desirable due to their promising applications, such as white LEDs; however, the development of highly efficient blue-emitting phosphors is still challenging. Here, we have quantitatively characterized the phosphorescence properties of the blue-emitting phosphor CaAl_2_O_4_:Eu^2+^, Nd^3+^ with various compositions and directly related these properties to the quality of its luminescence. We optimized the composition of the activator Eu^2+^ and the co-activator Nd^3+^, the doping conditions with alkaline earth metals, alkali metals, and Si to create crystallographic distortions and, finally, the flux conditions to find the best parameters for bright and persistent blue-emitting phosphors. Our research has identified several doping compositions with good to excellent performance, with which we have demonstrated bright and persistent phosphors with afterglow characteristics superior to those of conventional phosphors.

## Introduction

Phosphor materials have attracted much attention in applications such as electroluminescent displays, particularly white light emitting diodes (LEDs), and a large number of new phosphorescent materials have been developed in the last decade[1–7]. Among them, green-emitting ZnS:Cu phosphors have been used as long-lasting phosphorescent phosphors and applied in a variety of areas, including watches, clocks, traffic signs, emergency signage, and textile printing for signaling in the darkness[8, 9]. However, the applications of ZnS have been limited due to their short intrinsic decay time (1 h) and low emission intensity. Although doping with Co^2+^ enhances the emission intensity of ZnS:Cu phosphors, the incorporation of a large amount of a dopant into the host degrades the mechanical and physical properties of the host, particularly in the presence of moisture, which then becomes chemically unstable, limiting its application[5–7]. Another phosphor, YAG(yttrium aluminium garnet):Ce^3+^, is also a green- or yellow-emitting material; however, it also suffers from poor color rendition and high thermal quenching[10, 11]. To overcome this problem, Matsuzawa *et al*. have recently developed long lasting green-emitting phosphors of polycrystalline SrAl_2_O_4_ co-doped with Eu^2+^ and Dy^3+^, which resulted in improved lifetimes, intensity, and chemical stability over the previous phosphors[7]. Due to their higher chemical stability, the duration of the phosphorescence, and its high intensity, these materials could potentially replace the traditional ZnS-based phosphors, and further improvements have been recently achieved by many researchers[1–5].

Recently, white light emitting diodes (w-LEDs) are replacing conventional lighting products due to high efficiency, good material stability and long operation lifetime and phosphors are widely used as white LED sources[12]. A typical w-LEDs are fabricated by the combination of blue-emitting InGaN LEDs and yellow-emitting Y_3_Al_5_O_12_:Ce^3+^ (YAG:Ce^3+^) phosphor. However, this strategy has a number of disadvantages, such as high correlated color temperature (CCT > 4500 K) and low color rendering (CRI) index (R_a_ < 75)[13, 14]. An alternative strategy to generate white light is to coat near UV emitting LEDs with a mixture of high efficiency red, green and blue emitting phosphors which produces excellent CRI values and better color stability[15, 16]. However, it suffers from poor efficiency due to the large Stokes shift between emission and excitation of the near UV excitable phosphor. The eventual performance and efficiency of the w-LEDs strongly depends on the luminescence properties of the phosphors[17]. Therefore, it is highly desirable to develop highly efficient phosphors which can be excited by near UV LEDs. However, most of the improvements have been limited to green-emitting phosphors and the development of highly efficient blue-emitting phosphors is still challenging. The investigation of long-persistence blue-emitting phosphors is hence highly desirable, since the luminescence properties of blue-emitting phosphors can significantly affect the eventual performance of white LEDs[17].

Therefore, in this work, we have studied the effect of various doping compositions and impurities on the phosphorescence of blue-emitting calcium earth aluminate phosphor CaAl_2_O_4_:Eu^2+^, Nd^3+^ and improved its phosphorescence characteristics. The CaAl_2_O_4_:Eu^2+^ system is a blue emitting phosphor with an emission peak at 448 nm, analogous to the SrAl_2_O_4_:Eu^2+^ system[18–20]. It is known that the introduction of Nd^3+^ into the CaAl_2_O_4_:Eu^2+^ system boosts its phosphorescence intensity and lifetime, in a similar way as Dy^3+^ doping in the SrAl_2_O_4_:Eu^2+^ system[20]. However, the effects of Eu^2+^ and Nd^3+^ ions and/or native defects in the phosphorescence of CaAl_2_O_4_:Eu^2+^, Nd^3+^ remain unclear. In this paper, we present in detail the phosphorescence characteristics of CaAl_2_O_4_:Eu^2+^, Nd^3+^ crystals grown with various compositions by systematic investigations on the preparation, composition, structure, and luminescence properties, aiming at improving its phosphorescence characteristics.

## Results

### Composition of the activator and co-activator

**Fig 1:**
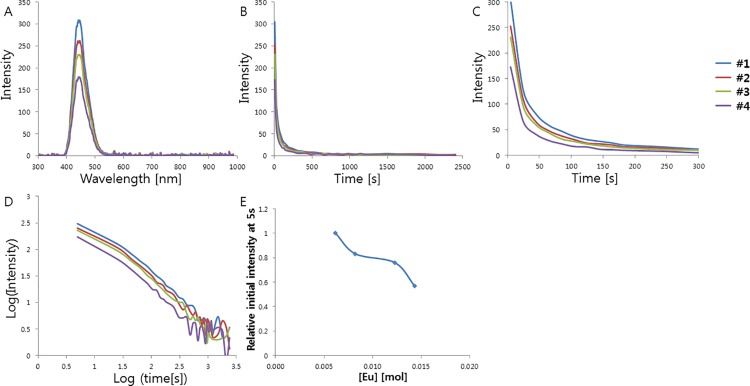
(A) Emission spectrum of CaAl_2_O_4_:Eu^2+^, Nd^3+^ crystals depending on Eu^2+^ concentration. (B) Decay curves depending on Eu^2+^ concentration. (C) Magnified views of the graph in (B). (D) Decay curves in log scale depending on Eu^2+^ concentration. (E) Relative initial intensity measured at 5s (relative values where the value of control sample #1 is 1.0) depending on Eu^2+^ concentration.

CaAl_2_O_4_ is known to have a monoclinic structure with three non-equivalent Ca sites[4]. When doped with Eu^2+^, those atoms occupy the only type of Ca sites that meets their size requirements[4]. Such Eu^2+^ ion doping boosts the intensity of the phosphorescence, and Eu^2+^ ions are typically used as the luminescence center and activator in the phosphor[20, 21]. Therefore, if the amount of Eu^2+^ incorporated in the host lattice is varied, the luminescence properties can be tuned owing to the significant changes in the local surroundings around a substituted site, such as the bond length and angle, as well as the point symmetry. In order to find the best conditions of Eu^2+^ doping for the development of a bright CaAl_2_O_4_:Eu^2+^, Nd^3+^ phosphor, we characterized the effect of the activator composition on the phosphorescence properties in an initial optimization. The samples were irradiated with 365 nm light for 5 min and the persistent luminescence was measured at room temperature for various Eu^2+^ concentrations ranging 0.006–0.014 mol. The phosphorescence spectra show the typical broadband emission resulting from the 5d → 4f transition in Eu^2+^, which is the transition between the crystal field components of the 4f^6^ 5d excited state configuration and the 4f^7^ ground state. These f–d transitions are known to be very sensitive to distortions in the crystal field[22]. The band position, shape, and width do not vary with the Eu^2+^ composition, while the persistence times vary greatly with the composition, indicating the same luminescent Eu^2+^ center at different Eu^2+^ compositions. An emission band is observed at 448 nm, at a shorter wavelength than that of SrAl_2_O_4_:Eu^2+^. As the radius of the alkaline earth ion (Ca^2+^, Sr^2+^, and Ba^2+^) increases, narrow emission bands are known to appear at progressively shorter wavelengths, which is consistent with our observations. The afterglow intensity was monitored for the emission peak at ~448 nm, which corresponds to the 5d → 4f transition in Eu^2+^. The decay curves of the afterglow intensity are shown in Fig 1. The curves are very similar for the different Eu^2+^ compositions and the only difference is the intensity. We further computed the lifetimes and decay rates of the emission at 448 nm by fitting them with three exponential components and different decay times[23]. The fitting results are presented in Table 1. The initial afterglow intensity measured after 5 s changes significantly with the Eu^2+^ concentration, while the decay times do not vary greatly with the composition. The decay curves show an initial rapid decay followed by long-persistence luminescence after removal of the UV light, consistent with the decay characteristics of phosphorescence. The observations suggest that the intensity decreases as the Eu^2+^ concentration increases, ~0.006 mol Eu^2+^ (per mol CaAl_2_O_4_:Eu^2+^, Nd^3+^) resulting in the brightest and longest emission, which is significantly lower than those for the optimized Eu^2+^ concentration of green-emitting phosphor SrAl_2_O_4_:Eu^2+^,Dy^3+^ (~0.935 mol Eu^2+^ per mol SrAl_2_O_4_:Eu^2+^,Dy^3+^). Owing to the larger size of Eu^2+^ (131 pm), it is relatively harder to introduce it in the Ca^2+^ sites of CaAl_2_O_4_ than in the similar-size Sr^2+^ sites (132 pm) of green-emitting SrAl_2_O_4_ crystals. Therefore, the afterglow of the phosphors is mainly influenced by the Eu^2+^ concentration and the host structure even when Eu^2+^ is used as both the activator and the luminescent center in those crystals. In addition, the increasing Eu^2+^ concentration could cause significant changes in the local surroundings, such as point symmetry, bond length, bond angle around a substituted site, interrupting the phosphorescence mechanism in CaAl_2_O_4_: Eu^2+^, Nd^3+^.

**Table 1:** Various nominal activator(Eu^2+^) compositions of the CaAl_2_O_4_:Eu^2+^, Nd^3+^ crystals and the calculated decay times of the phosphorescence from the CaAl_2_O_4_:Eu^2+^, Nd^3+^ crystals doped with various Eu^2+^ concentrations. Decay times were calculated based on the three exponential components ($I=a\;*\;e^{-\frac{t}{t1}}+b\;*\;e^{-\frac{t}{t2}}+c\;*\;e^{-\frac{t}{t3}}$) by a curve fitting technique.

Sample	#1	#2	#3	#4
Mol of Eu	CaAl_2_O_4_:Eu_0.006_,Nd	CaAl_2_O_4_:Eu_0.008_,Nd	CaAl_2_O_4_:Eu_0.012_,Nd	CaAl_2_O_4_:Eu_0.014_,Nd
**t1[s]**	202.4	164.1	220	132.6
**t2[s]**	30.74	25.15	31.26	25.04
**t3[s]**	0.5419	0.6566	0.9798	0.3373
**a**	44.17	42.53	28.79	29.15
**b**	145	131.6	107.3	79.45
**c**	0.9492	0.8655	0.6647	0.9011

**Fig 2:**
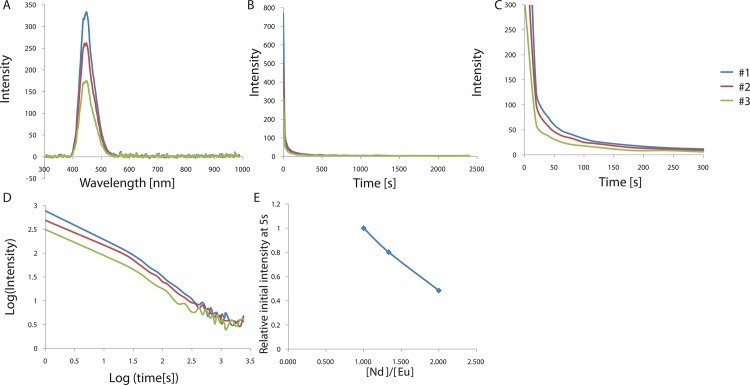
(A) Emission spectrum of CaAl_2_O_4_:Eu^2+^, Nd^3+^ crystals depending on [Nd^3+^]/[Eu^2+^] ratio. (B) Decay curves depending on [Nd^3+^]/[Eu^2+^] ratio. (C) Magnified views of the graph in (B). (D) Decay curves in log scale depending on [Nd^3+^]/[Eu^2+^] concentration. (E) Relative initial intensity measured at 5s (relative values where the value of control sample #1 is 1.0) depending on [Nd^3+^]/[Eu^2+^] concentration.

**Fig 3:**
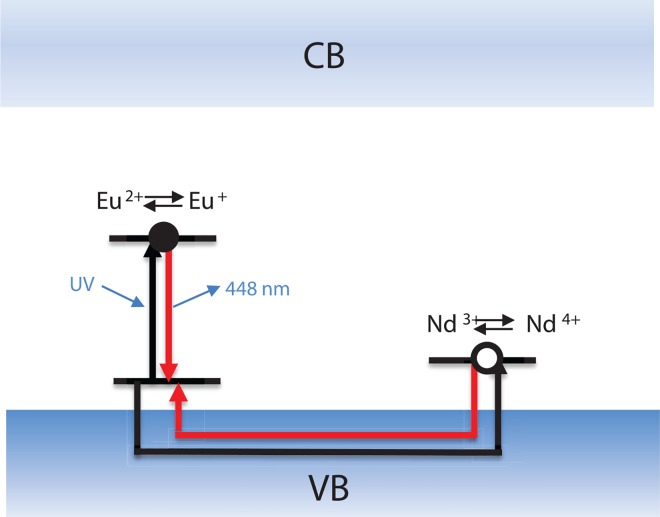
Energy level diagram for CaAl2O4: Eu^2+^, Nd^3+^ Phosphor.

Based on this result, we also evaluated the effect of different activator and co-activator compositions on the phosphorescence intensity. It is known that Nd^3+^ doping produces extremely brighter and longer blue phosphorescence than Dy^3+^ doping at room temperature in blue-emitting CaAl_2_O_4_: Eu^2+^[20, 21]. Its introduction is analogous to that of Dy^3+^ as an auxiliary activator in green-emitting SrAl_2_O_4_:Eu^2+^ to produce remarkably intense phosphorescence[7, 23–27]. Previous studies have reported that Nd^3+^ doping results in stronger phosphorescence at room temperature than Dy^3+^ doping in CaAl_2_O_4_:Eu^2+^, indicating that the local environment may affect the trap depth made by the co-dopant[20]. In our previous study, we reported that the optimum concentration of the activator Eu^2+^ was ~0.935 mol (per mol of SrAl_2_O_4_) and the optimum Dy^3+^/Eu^2+^ ratio was ~2.4[28]. In a similar way, we tested different Nd^3+^/Eu^2+^ ratios by varying the concentration of Nd^3+^ at a constant Eu^2+^ concentration (0.006 mol per mol CaAl_2_O_4_:Eu^2+^, Nd^3+^) to find the optimal concentration of Nd^3+^. The phosphor samples were prepared by firing mixtures of CaCO_3_, Al_2_O_3_, Eu_2_O_3_, SiO_2_, and small quantities of H_3_BO_3_ as a flux in a reducing atmosphere at 1300°C for 3–5 h and measuring the phosphorescence. The afterglow curves measured at 448 nm are shown in Fig 2. The Nd^3+^/Eu^2+^ ratio was varied in the range from 1 to 2.4 and the results are shown in Fig 2 and Table 2. The results indicate that the intensity decreases as the Nd^3+^/Eu^2+^ ratio increases, and the Nd^3+^/Eu^2+^ ratio of 1 results in the brightest and longest emission. This optimum concentration of Nd^3+^ (~0.006 mol per mol CaAl_2_O_4_:Eu^2+^, Nd^3+^) is much lower than the optimum concentration of Dy^3+^ (~2.244 mol per mol CaAl_2_O_4_:Eu^2+^, Nd^3+^) in SrAl_2_O_4_:Eu^2+^, Dy^3+^ reported in a previous study, which could be explained by the difference in the solubility of the two ions in the structure[28]. Since the ion radius of Dy^3+^ is smaller than that of Nd^3+^, Dy^3+^ is more soluble within the system than Nd^3+^[7]. Therefore, a higher concentration of Dy^3+^ can be incorporated into the structure, forming a relatively higher number of trapping levels and resulting in brighter phosphorescence in the SrAl_2_O_4_:Eu^2+^ system compared to the case of Nd^3+^ doping in CaAl_2_O_4_:Eu^2+^. Doping with a higher concentration of Nd^3+^ (reaching levels over the solubility limit) may result in the production of the by-product NdAlO_3_, disrupting the phosphorescence process. The role of Nd^3+^ doping in CaAl_2_O_4_:Eu^2+^ can be explained by hole traps in the structure, similar to what happens with Dy^3+^ in SrAl_2_O_4_:Eu^2+^[23, 29]. Since Nd^3+^ and Dy^3+^ have relatively low 4f–5d transition energies and high charge-transfer energies, they can act as hole traps[23, 29]. These holes migrate to the excited Eu^2+^ centers where they are captured, followed by recombination. Phosphorescence is caused by this trapping of photo-generated holes and/or electrons, which, following a delayed radiative return after recombination of the charge carriers, causes luminescence. Therefore, phosphorescence is considered as thermo-luminescence with de-trapping at room temperature, and local distortions around the co-dopant ions seem to affect the trap depth. The trapping level of the CaAl_2_O_4_:Eu^2+^, Nd^3+^ phosphor is known to be located a little shallower than that of the SrAl_2_O_4_:Eu^2+^, Dy^3+^ phosphor, resulting in relatively shorter phosphorescence, which agrees with our observations. However, the trapping level of the CaAl_2_O_4_:Eu^2+^, Nd^3+^ phosphor is probably not so shallow to show a fast decay that does not last for long, but deep enough to show long phosphorescence at room temperature. The de-trapping mechanism in CaAl_2_O_4_:Eu^2+^, Nd^3+^ is described in Fig 3. In this mechanism, Nd^3+^ acts as a hole trap and the holes move to the excited state of Eu^2+^. After capturing, recombination occurs, followed by phosphorescence. Therefore, local distortions around co-dopant ions seem to affect the trap depth, and hence, optimization of the activator and co-activator composition is important to produce high phosphorescence intensity.

**Table 2:** Various nominal activator(Eu^2+^) and co-activator(Nd^3+^) compositions of the CaAl_2_O_4_:Eu^2+^, Nd^3+^ crystals and the calculated decay times of the phosphorescence from the CaAl_2_O_4_:Eu^2+^, Nd^3+^ crystals doped with various [Nd^3+^]/[Eu^3+^] ratios. Decay times were calculated based on the three exponential components($I=a\;*\;e^{-\frac{t}{t1}}+b\;*\;e^{-\frac{t}{t2}}+c\;*\;e^{-\frac{t}{t3}}$) by a curve fitting technique.

Sample	#1	#2	#3
Mol of Nd	CaAl_2_O_4_:Eu_0.006_,Nd_0.006_	CaAl_2_O_4_:Eu_0.006_,Nd_0.008_	CaAl_2_O_4_:Eu_0.006_,Nd_0.012_
**t1[s]**	**171.3**	**159**	**193.5**
**t2[s]**	**25.16**	**22**	**29.18**
**t3[s]**	**0.2395**	**0.2581**	**0.2675**
**a**	**41.91**	**36.24**	**16.93**
**b**	**172.9**	**147.4**	**77.98**
**c**	**0.9816**	**0.9809**	**0.9806**

### Doping with impurities

In blue-emitting CaAl_2_O_4_:Eu^2+^, Nd^3+^, the Eu^2+^ ions usually act as the luminescence centers and the transitions between the 4f^7^ ground state and the crystal field components of the 4f^6^ 5d excited state are responsible for the broad emission spectrum of Eu^2+^, as previously explained[20]. Such f–d transitions are known to be very sensitive to distortions of the crystal field in the luminescent host of alkaline earth silicates[22]. Therefore, if this stable host structure changes and crystallographic distortions occur by substitution or impurities, the crystal field environment of the rare earth ions in the host structure is influenced, affecting the trap depth and, finally, the characteristics of the phosphorescence. In order to create such crystallographic distortions and boost the luminescence in CaAl_2_O_4_:Eu^2+^, Nd^3+^, we substituted Ca^2+^ with alkali metal or alkaline earth metal ions of various sizes, or substituted the Al^3+^ cations with Si^4+^. These substitutions may lead to less forbidden transitions, thereby enhancing the phosphorescence.

### Doping with impurities—Alkaline earth metal doping

**Fig 4:**
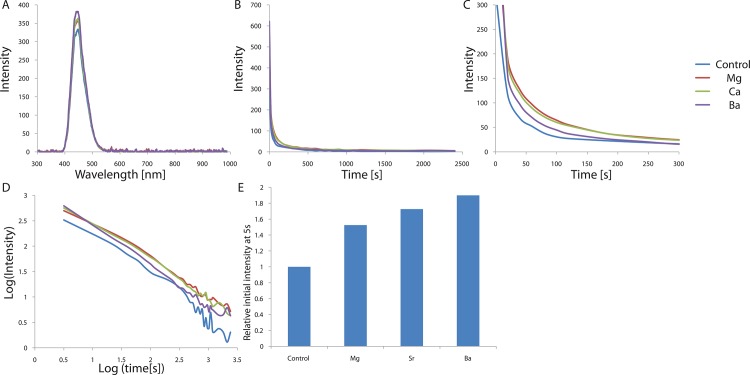
(A) Emission spectrum of CaAl_2_O_4_:Eu^2+^, Nd^3+^ crystals depending on alkali earth metal ion doping. (B) Decay curves depending on alkali earth metal ion doping. (C) Magnified views of the graph in (B). (D) Decay curves in log scale depending on alkali earth metal ion doping. (E) Relative initial intensity measured at 5s (relative values where the value of control sample is 1.0) depending on alkali earth metal ion doping.

As an initial doping test, we substituted Ca^2+^ in the calcium aluminate phosphor with alkaline earth metal ions of different sizes, in order to create crystallographic distortions in the host structure and boost the phosphorescence. High purity chemical reagents CaCO_3_, Al_2_O_3_, Eu_2_O_3_, SiO_2_, MCO_3_ (M = Sr^2+^, Mg^2+^, and Ba^2+^) and small quantities of H_3_BO_3_ were used as starting materials, and the dried powder mixtures were fired in the furnace at 1300°C for 3–5 h. The phosphors were irradiated with 365-nm light for 5 min at room temperature and the afterglow spectra, decay curves, and fitting results from different phosphors and doping samples are shown in Fig 4 and Table 3. Since the ionic radii of alkaline earth metals decrease from Ba^2+^ to Mg^2+^ in the body-centered cubic crystal structure (Ba^2+^: 149 pm, Sr^2+^: 132 pm, Ca^2+^:114 pm, Mg^2+^: 86 pm), we expected different orders of break in the symmetry of the host crystal structure depending on the size difference (relative to Ca^2+^), resulting in expansion or shrinkage of the structure. The wavelength position, band shape, and bandwidth of the afterglow did not change with the addition of any alkaline earth doping, as shown in Fig 4, indicating that the emitting centers are still the Eu^2+^ ions. The decay times of the CaAl_2_O_4_:Eu^2+^, Nd^3+^ crystals grown from different starting compositions have similar values; however, the intensity of the phosphorescence after illumination is different (Table 3). As expected, alkali metal doping significantly increases the luminescence up to 190% of the initial value, as compared to that observed for the non-doped crystal, probably due to the distorted crystal structure leading to less forbidden transitions. The largest alkaline metal, Ba^2+^, exhibits the largest increase in initial luminescence at 5 s, probably due to the largest size difference with respect to Ca^2+^, while the smallest alkaline metal, Mg^2+^, displays the smallest increase in initial luminescence at 5 s due to the smallest size difference with Ca^2+^.

**Table 3:** Nominal compositions of the CaAl_2_O_4_:Eu^2+^, Nd^3+^ crystals doped with different Alkaline Earth metal ions and the calculated decay times of the phosphorescence from the CaAl_2_O_4_:Eu^2+^, Nd^3+^ crystals doped with various alkaline earth metals. Decay times were calculated based on the three exponential components ($I=a\;*\;e^{-\frac{t}{t1}}+b\;*\;e^{-\frac{t}{t2}}+c\;*\;e^{-\frac{t}{t3}}$) by a curve fitting technique.

Sample	#1	#2	#3	#4
Alkaline Earth Metal	CaAl_2_O_4_:Eu,Nd	CaAl_2_O_4_:Eu,Nd,Mg	CaAl_2_O_4_:Eu,Nd,Sr	CaAl_2_O_4_:Eu,Nd,Ba
**t1[s]**	285.7	235.8	209.1	217.8
**t2[s]**	25.28	34.06	29.2	29.54
**t3[s]**	0.7052	0.405	0.3382	0.3097
**a**	37.46	64.91	71.9	48.11
**b**	150	202.6	194.6	191.1
**c**	0.8194	0.9499	0.9309	0.915

**Fig 5:**
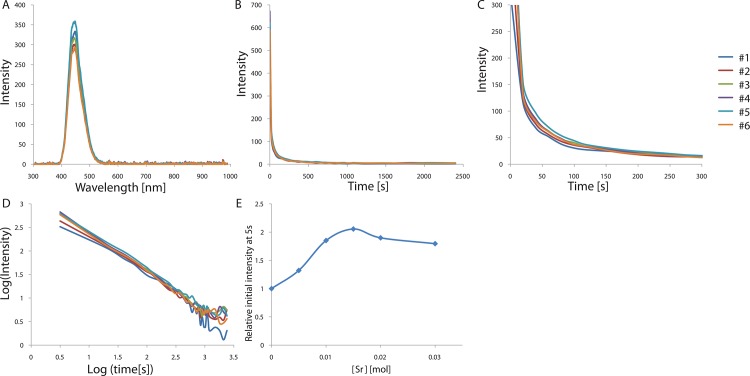
(A) Emission spectrum of CaAl_2_O_4_:Eu^2+^, Nd^3+^ crystals curves depending on Sr^2+^ concentration. (B) Decay curves depending on Sr^2+^ concentration. (C) Magnified views of the graph in (B). (D) Decay curves in log scale depending on Sr^2+^ concentration. (E) Relative initial intensity measured at 5s (relative values where the value of control sample #1 is 1.0) depending on Sr^2+^ concentration.

In order to optimize further the conditions, we next tested different concentrations of alkaline metal doping. We chose Sr^2+^ doping, rather than Ba^2+^ doping, since Sr^2+^ doping exhibits a slower decay after 10 s than Ba^2+^ doping. Various concentrations of SrCO_3_ from 0 to 0.03 mol (per mol of CaAl_2_O_4_:Eu^2+^, Nd^3+^) were tested and the results are shown in Fig 5 and Table 4. Again, the shape and bandwidth of the UV-excited luminescence did not change at different concentrations of SrCO_3_, indicating again the Eu^2+^ centers. We found that all of the phosphors doped with SrCO_3_ displayed enhanced phosphorescence up to 206% of the initial value, as compared to that observed for the non-doped crystal. The strongest initial phosphorescence was observed with 0.015 mol of SrCO_3_ (per mol of CaAl_2_O_4_:Eu^2+^, Nd^3+^). Concentrations below 0.015 mol of SrCO_3_ may not be enough to enhance the electronic transitions of Eu^2+^, while concentrations above 0.015 mol of SrCO_3_ may disrupt the overall crystal structure, decreasing the phosphorescence.

**Table 4:** Nominal compositions of the CaAl_2_O_4_:Eu^2+^, Nd^3+^ crystals doped with different Sr^2+^ concentrations and the calculated decay times of the phosphorescence from the CaAl_2_O_4_:Eu^2+^, Nd^3+^ crystals doped with various Sr^2+^ concentrations. Decay times were calculated based on the three exponential components ($I=a\;*\;e^{-\frac{t}{t1}}+b\;*\;e^{-\frac{t}{t2}}+c\;*\;e^{-\frac{t}{t3}}$) by a curve fitting technique.

Sample	#1	#2	#3	#4	#5	#6
Mol of Sr	CaAl_2_O_4_:Eu,Nd	CaAl_2_O_4_:Eu,Nd,Sr_0.005_	CaAl_2_O_4_:Eu,Nd,Sr_0.010_	CaAl_2_O_4_:Eu,Nd,Sr_0.015_	CaAl_2_O_4_:Eu,Nd,Sr_0.020_	CaAl_2_O_4_:Eu,Nd,Sr_0.030_
**t1[s]**	285.7	159.5	182	175.4	217.8	183.4
**t2[s]**	25.28	22.82	26.25	26.42	29.54	28.02
**t3[s]**	0.2077	0.3476	0.2959	0.2927	0.3097	0.2954
**a**	37.46	52.94	51.35	50.35	48.11	48.43
**b**	150	161.5	161.4	173.4	191.1	154.5
**c**	0.7052	0.9284	0.9085	0.9097	0.915	0.9087

### Doping with impurities—Alkali metal doping

**Fig 6:**
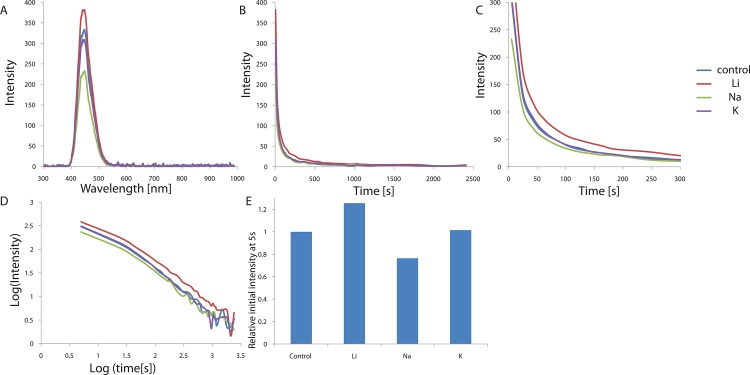
(A) Emission spectrum of CaAl_2_O_4_:Eu^2+^, Nd^3+^ crystals depending on alkali metal ion doping. (B) Decay curves depending on alkali metal ion doping. (C) Magnified views of the graph in (B). (D) Decay curves in log scale depending on alkali metal ion doping. (E) Relative initial intensity measured at 5s (relative values where the value of control sample is 1.0) depending on alkali metal ion doping.

To further break the centro-symmetry of the structure, we tried to substitute Ca^2+^ with alkali metals. Doping with alkali metals is expected to result in two effects: a decrease in cation vacancies due to charge differences, and changes in the crystal structure symmetry due to size differences. These two effects may cause the corresponding shrinkage or expansion of the host structure and changes in the formation of hole traps, thereby resulting in the change of the afterglow characteristics. The powder materials SrCO_3_, Al_2_O_3_, Nd_2_O_3_, Eu_2_O_3_, and M_2_CO_3_ (M = Li, Na, and K) were weighed out and mixed according to the mole ratios of the elements in the final product, and boric acid was added as a flux to prepare the polycrystalline CaAl_2_O_4_: Eu^2+^, Nd^3+^. The compounds were pressed into pellets, followed by sintering into ceramics at 1300°C for 3–5 h in a N_2_ / H_2_ reducing atmosphere. The final products were irradiated with 365-nm UV light for 5 min, and the phosphorescence and afterglow curves were measured at 448 nm, as shown in Fig 6 and Table 5. The band position, shape, and width of the UV-excited luminescence were found to be identical, indicating the same Eu^2+^ centers in the different compounds doped with different alkali metals. However, the phosphorescence intensities varied significantly with the alkali metal doping, although the decay times of the CaAl_2_O_4_:Eu^2+^, Nd^3+^ doped with different alkali metal ions were almost similar. Only Li^+^ doping showed an increase in the initial phosphorescence intensity measured 5 s after removing the UV-light, and Na^+^ and K^+^ doping showed similar or lower intensity compared to the non-doped compounds, suggesting changes in the afterglow intensity by the different sized-alkali metal doping. The ionic radii of the alkali metals decrease smoothly from K^+^ to Li^+^ (Li^+^: 90 pm, Na^+^: 116 pm, K^+^: 152 pm, Ca^2+^: 114 pm); Na^+^ and K^+^ are larger than Ca^2+^, while Li^+^ is smaller than Ca^2+^. Therefore, the Na^+^ and K^+^ ions may not be able to enter the cation vacancies since they are larger than Ca^2+^, thus decreasing the number of cation vacancies and hole traps. The results suggest that Na^+^ or K^+^ co-doping likely quenches the afterglow luminescence intensity efficiently due to a decrease in the number of cation vacancies.

**Table 5:** Nominal compositions of the CaAl_2_O_4_:Eu^2+^, Nd^3+^ crystals doped with different alkali metals and the calculated decay times of the phosphorescence from the CaAl_2_O_4_:Eu^2+^, Nd^3+^ crystals doped with various alkali metals. Decay times were calculated based on the three exponential components($I=a\;*\;e^{-\frac{t}{t1}}+b\;*\;e^{-\frac{t}{t2}}+c\;*\;e^{-\frac{t}{t3}}$) by a curve fitting technique.

Sample	#1	#2	#3	#4
Alkali Metal	CaAl_2_O_4_:Eu,Nd	CaAl_2_O_4_:Eu,Nd,Li_0.002_	CaAl_2_O_4_:Eu,Nd,Na_0.002_	CaAl_2_O_4_:Eu,Nd,K_0.002_
**t1[s]**	294.8	246.6	191.6	283.1
**t2[s]**	52.81	34.46	33.45	47.04
**t3[s]**	0.07818	0.08444	0.628	0.1656
**a**	28.04	58.17	38.71	31.18
**b**	115.9	247.1	140.3	135.2
**c**	0.1524	0.004634	0.3993	0.1622

**Fig 7:**
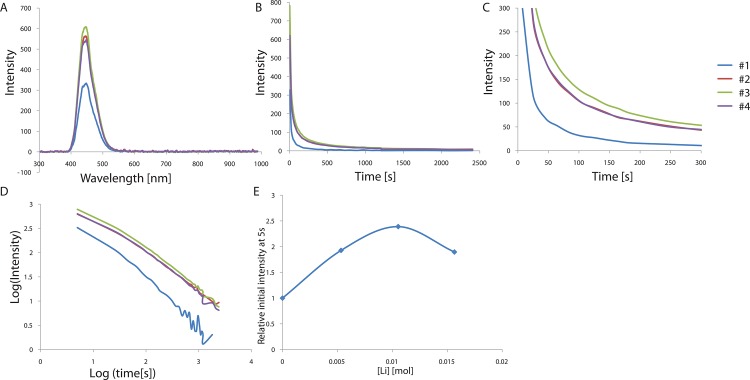
(A) Emission spectrum of CaAl_2_O_4_:Eu^2+^, Nd^3+^ crystals depending on Li^+^ concentration. (B) Decay curves depending on Li^+^ concentration. (C) Magnified views of the graph in (B). (D) Decay curves in log scale depending on Li^+^ concentration. (E) Relative initial intensity measured at 5s (relative values where the value of control sample #1 is 1.0) depending on Li+ concentration.

Next, in order to take full advantage of Li^+^ doping, we also tested various concentrations of Li^+^ doping from 0 mol to 0.016 mol (per mol of CaAl_2_O_4_:Eu^2+^, Nd^3+^). Doping with different concentrations of Li^+^ shows a similar band position, shape, and width, but different initial phosphorescence intensity (Fig 7 and Table 6). We found that all of the Li+ doping with different concentration of the Li^+^(0.005~0.016 mol) enhance the phosphorescence from 190% up to 239% of the initial value, as compared to that observed for the non-doped crystal. The optimal concentration of Li^+^ was 0.010 mol (per mol of CaAl_2_O_4_:Eu^2+^, Nd^3+^), which was similar to the previously reported optimal concentration of Li^+^ in the SrAl_2_O_4_:Eu^2+^, Dy^3+^. This concentration is likely enough to enter into the cation vacancies, enhancing the electronic transition of Eu^2+^ but not too high to disrupt the overall crystal structure.

**Table 6:** Nominal compositions of the CaAl_2_O_4_:Eu^2+^, Nd^3+^ crystals doped with different Li^+^ concentrations and the calculated decay times of the phosphorescence from the CaAl_2_O_4_:Eu^2+^, Nd^3+^ crystals doped with various Li^+^ concentrations. Decay times were calculated based on the three exponential components($I=a\;*\;e^{-\frac{t}{t1}}+b\;*\;e^{-\frac{t}{t2}}+c\;*\;e^{-\frac{t}{t3}}$) by a curve fitting technique.

Sample	#1	#2	#3	#4
Mol of Li	CaAl_2_O_4_:Eu,Nd	CaAl_2_O_4_:Eu,Nd,Li_0.005_	CaAl_2_O_4_:Eu,Nd,Li_0.010_	CaAl_2_O_4_:Eu,Nd,Li_0.016_
**t1[s]**	287.2	284.1	280.4	331.3
**t2[s]**	46.2	45.18	44.95	49.54
**t3[s]**	0.03054	0.8594	0.4899	0.682
**a**	24.62	89.85	114.5	82.79
**b**	120.5	263.3	317.6	262.8
**c**	0.4253	0.4799	0.4799	0.7127

### Doping with impurities—Si^4+^ doping

**Fig 8:**
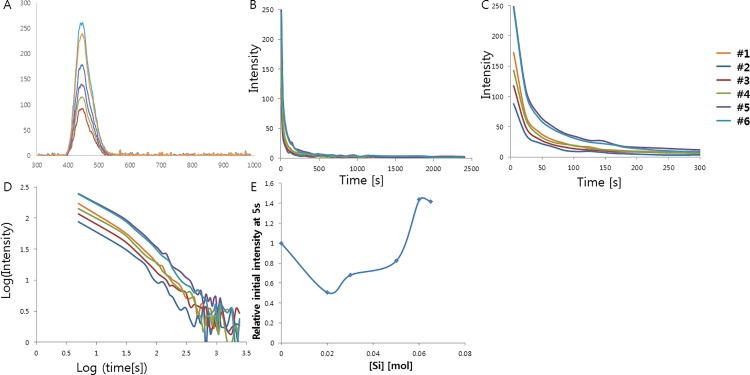
(A) Emission spectrum of CaAl_2_O_4_:Eu^2+^, Nd^3+^ crystals depending on Si^4+^ concentration. (B) Decay curves depending on Si^4+^ concentration. (C) Magnified views of the graph in (B). (D) Decay curves in log scale depending on Si^4+^ concentration. (E) Relative initial intensity measured at 5s (relative values where the value of control sample #1 is 1.0) depending on Si^4+^ concentration.

Next, we carried out the experiment of doping with SiO_2_ in order to substitute Al^3+^ with Si^4+^ in the CaAl_2_O_4_:Eu^2+^, Nd^3+^ crystalline structure. Doping with SiO_2_ is expected to cause not only the creation of cation vacancies, but also the shrinkage of the crystal structure since the size of Si^4+^ (~40 pm) in tetrahedral mode is smaller than the size of Al^3+^ (53 pm). If optimal, the cation vacancies, which act as hole traps, and breaking the symmetry of the crystal structure by shrinkage or expansion of the crystal structure contribute to boost the phosphorescence intensity and increase the lifetime of the phosphorescence. To figure out the effect of Si^4+^ doping on CaAl_2_O_4_:Eu^2+^, Nd^3+^, various concentrations of SiO_2_ were tested, ranging from 0 mol to 0.065 mol. The compounds were prepared in the same way as the previous experiments, and the decay curves of the afterglow and the fitting results are shown in Fig 8 and Table 7. The band position, shape, and width of the UV-excited spectra appear similar, implying the same luminescent centers. However, the intensities of the afterglow appear different at the different concentrations of Si doping. Interestingly, the initial afterglow intensities were lower than that observed from the non-doped crystal at concentrations of Si^4+^ below 0.05 mol, whereas the initial afterglow intensities became brighter at concentrations of Si^4+^ above 0.06 mol. This observation could be explained by two different effects from Si^4+^ doping in the crystal structures: a shrinking effect caused by the smaller size of Si^4+^ and an expansion effect caused by the creation of cation vacancies. If these two effects cancel out each other, there is no or a minimal boost effect on the phosphorescence. It may be the case when the concentration of Si^4+^ is lower than 0.05 mol, and additional disruption of the overall crystal structure likely decreases the phosphorescence intensity. However, if these two effects are synergic and the hole traps with the optimal depth are created by cation vacancies, the phosphorescence is enhanced. This may be the case when the concentration of Si^4+^ is higher than 0.06 mol. Finally, we found that the optimal concentration of Si^4+^ is 0.06 mol, enhancing the phosphorescence intensity up to 144% that of the original value for the non-doped crystal.

**Table 7:** Nominal compositions of the CaAl_2_O_4_:Eu^2+^, Nd^3+^ crystals doped with different Si^4+^ concentrations and the calculated decay times of the phosphorescence from the CaAl_2_O_4_:Eu^2+^, Nd^3+^ crystals doped with various Si^4+^ concentrations. Decay times were calculated based on the three exponential components ($I=a\;*\;e^{-\frac{t}{t1}}+b\;*\;e^{-\frac{t}{t2}}+c\;*\;e^{-\frac{t}{t3}}$) by a curve fitting technique.

Sample	#1	#2	#3	#4	#5	#6
Mol of Si	CaAl_2_O_4_:Eu,Nd	CaAl_2_O_4_:Eu,Nd,Si_0.020_	CaAl_2_O_4_:Eu,Nd,Si_0.030_	CaAl_2_O_4_:Eu,Nd,Si_0.050_	CaAl_2_O_4_:Eu,Nd,Si_0.060_	CaAl_2_O_4_:Eu,Nd,Si_0.065_
**t1[s]**	132.6	113.9	207.7	149.6	171.1	137
**t2[s]**	25.04	29.61	27.97	21.15	28.74	24.52
**t3[s]**	0.336	0.566	0.426	0.385	0.562	0.6541
**a**	29.15	15.4	14.33	27.08	43.46	48.8
**b**	79.46	37.49	61.81	83.47	119	111.5
**c**	0.9006	0.7582	0.7413	0.8444	0.9549	0.8646

### Optimization of the flux

**Fig 9:**
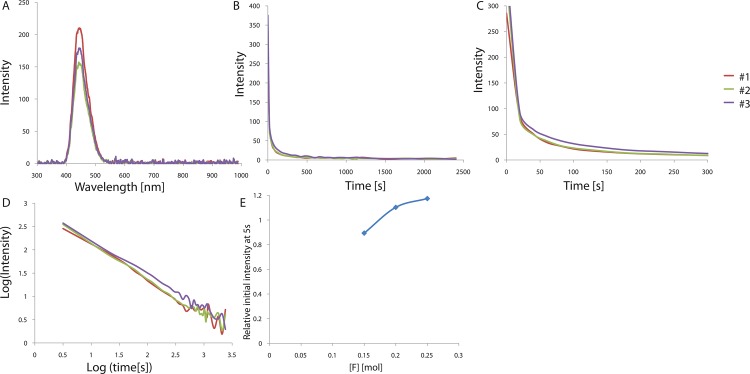
(A) Emission spectrum of CaAl_2_O_4_:Eu^2+^, Nd^3+^ crystals curves depending on H_3_BO_3_ concentration. (B) Decay curves depending on H_3_BO_3_ concentration. (C) Magnified views of the graph in (B). (D) Decay curves in log scale depending on H_3_BO_3_ concentration. (E) Relative initial intensity measured at 5s (relative values where the value of control sample #1 is 1.0) depending on H_3_BO_3_ concentration.

Further enhancements can be achieved by optimizing the flux conditions. In this study, H_3_BO_3_ was used as the flux, and we tested various concentrations of H_3_BO_3_ to find its optimal concentration. At high concentrations of H_3_BO_3_, it was hard to remove the hardened final product from the crucibles after firing and, even when it was removed from the crucibles, the final product was too hard to be ground in the mortar. Therefore, we tested concentrations of H_3_BO_3_ from 0.15 mol to 0.25 mol per mol of CaAl_2_O_4_:Eu^2+^, Nd^3+^. From the measurements (Fig 9 and Table 8), we found that the phosphorescence slightly increased with the concentration of H_3_BO_3_. The optimal concentration of H_3_BO_3_ was found to be 0.25 mol, since an excess of H_3_BO_3_ may form trigonal planar BO_3_ by-product units, probably disrupting the phosphorescence mechanism.

**Table 8:** Nominal compositions of the CaAl_2_O_4_:Eu^2+^, Nd^3+^ crystals synthesized with different H_3_BO_3_ concentrations and the calculated decay times of the phosphorescence from the CaAl_2_O_4_:Eu^2+^, Nd^3+^ crystals doped with various Si^4+^ concentrations. Decay times were calculated based on the three exponential components ($I=a\;*\;e^{-\frac{t}{t1}}+b\;*\;e^{-\frac{t}{t2}}+c\;*\;e^{-\frac{t}{t3}}$) by a curve fitting technique.

Sample	#1	#2	#3
Mol of H_3_BO_3_	0.15	0.20	0.25
**t1[s]**	156.3	174.6	203.6
**t2[s]**	24.63	28.23	27.73
**t3[s]**	0.3777	0.2626	0.3132
**a**	28.17	28.14	37.43
**b**	114.5	94.67	100.6
**c**	0.9703	0.9807	0.9036

## Discussion

Here, we have described the systematic characterization of the phosphorescence properties of CaAl_2_O_4_:Eu^2+^, Nd^3+^ synthesized with various compositions to develop bright and persistent blue-emitting phosphors. In an initial test, the activator and co-activator compositions were optimized and then, we tried to substitute Ca^2+^ with alkali metal or alkaline earth metal ions of various sizes, or substituted Al^3+^ with Si^4+^ to create crystallographic distortions and boost the luminescence of CaAl_2_O_4_:Eu^2+^, Nd^3+^. In general, the band position, shape, and width did not vary, while the persistence times and intensities varied greatly with the different compositions, indicating the same luminescent Eu^2+^ centers are present in the different compositions we tried. Therefore, we quantitatively characterized the afterglow intensity to find the optimized conditions for bright and persistent blue-emitting phosphors. In the composition studies on the activator Eu^2+^ and the co-activator Nd^3+^, ~0.006 mol Eu^2+^ (per mol CaAl_2_O_4_:Eu^2+^, Nd^3+^) and a Nd^3+^/Eu^2+^ ratio of 1 resulted in the brightest and longest emission. These are much lower concentrations than the optimum concentration of Eu^2+^ (~0.935 mol Eu^2+^ per mol SrAl_2_O_4_:Eu^2+^, Dy^3+^) and Dy^3+^ (~2.244 mol per mol SrAl_2_O_4_:Eu^2+^, Dy^3+^) in the green-emitting SrAl_2_O_4_:Eu^2+^, Dy^3+^ phosphor reported in our previous study, probably owing to the low solubility of Eu^2+^ and Nd^3+^ in the CaAl_2_O_4_ crystal due to their relatively large sizes[28]. The different compositions of Eu^2+^ and Nd^3+^ in CaAl_2_O_4_:Eu^2+^, Nd^3+^ resulted in big differences in the afterglow intensity and hence, the optimization of the activator and co-activator composition was found to be important towards a high phosphorescence intensity. In the alkaline earth metal doping test, the alkaline metal doping achieved significant enhancements of the luminescence up to 190% of the initial value, as compared to that observed for the non-doped crystal, probably due to the distorted crystal structure with alkaline earth metal doping leading to less forbidden transitions. Among them, the largest alkaline metal, Ba^2+^, showed the largest increase in initial luminescence, while the smallest alkaline metal, Mg^2+^, showed the smallest increase in initial luminescence. When we varied the concentration of SrCO_3_, the phosphorescence was enhanced up to 206% of the initial value with 0.015 mol of SrCO_3_ (per mol of CaAl_2_O_4_:Eu^2+^, Nd^3+^), as compared to that observed for the non-doped crystal. In the alkali metal doping test, only Li^+^ doping showed an increase in the initial phosphorescence intensity, while Na^+^ and K^+^ doping showed a similar or lower intensity compared to the non-doped compound. When we varied the concentration of Li^+^ to find the optimal value, the phosphorescence was enhanced up to 239% of the initial value with 0.010 mol per mol of CaAl_2_O_4_:Eu^2+^, Nd^3+^, as compared to that observed for the non-doped crystal. From the Si doping test, we found that the phosphorescence intensity could be enhanced up to 144% of the original value at the optimal concentration of Si^4+^ (0.06 mol). Lastly, the flux was also found to affect the phosphorescence. The phosphorescence could be slightly increased as the concentration of H_3_BO_3_ increased; however, amounts larger than 0.25 mol of H_3_BO_3_ make the compound too hard and difficult to remove from the crucibles after firing and grinding in the mortar.

**Fig 10:**
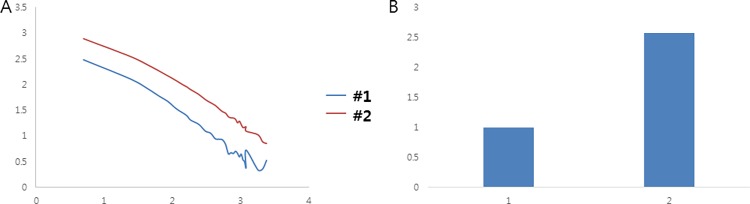
(A) Decay curves in log scale of the best optimized CaAl_2_O_4_:Eu^2+^, Nd^3+^ crystals(#2) compared with the control CaAl_2_O_4_:Eu^2+^, Nd^3+^ crystals which is doped with optimized Eu^2+^ and Nd^3+^(#1). (B) Relative initial intensity measured at 5s (relative values where the value of control sample #1 is 1.0)

Finally, we found that our combined optimized condition which are doping with ~0.006 mol Eu^2+^, ~0.006 mol Nd^3+^, 0.015 mol of SrCO_3_, 0.010 mol Li^+^, 0.06 mol Si^4+^ and 0.25 mol of H_3_BO_3_ (per 1mol SrAl_2_O_4_:Eu^2+^, Dy^3+^) boosts the phosphorescence intensity to 257% of the initial value which is doping with ~0.006 mol Eu^2+^, ~0.006 mol Nd^3+^(Fig 10). This investigation is expected to provide a guideline for the synthesis of bright and long persistent blue-emitting phosphors, and facilitate the application of persistent phosphors with afterglow characteristics superior to those of conventional phosphors. Although the detailed mechanism of the doping effects on the persistent luminescence remains an open question, we note that the role of lattice defects in CaAl_2_O_4_:Eu^2+^, Nd^3+^ as traps are likely of great importance for the persistence of the luminescence. Further works utilizing different experimental spectroscopic and other techniques such as XRD and microscopy regarding phase and purity confirmation would be valuable to explore the details of the phosphorescence mechanism in CaAl_2_O_4_:Eu^2+^, Nd^3+^, the co-doping effects and the optimization of alkaline earth metals and alkali metals to further enhance the phosphorescence efficiency. This blue-emitting material is expected to be used as a novel phosphor with numerous applications in not only white LEDs, but also in the areas of energy saving and safety improvement.

## Methods

The details of our experimental methods have been described previously[28]. Briefly, all CaAl_2_O_4_:Eu^2+^, Nd^3+^ powder samples were synthesized by a high-temperature solid-state reaction. High-purity SrCO_3_, Eu_2_O_3_ (Rhône-Poulenc, 99.99%), Nd_2_O_3_, Al_2_O_3_, MCO_3_ (M = Ca, Sr, and Ba; Merck, >99.0%), and SiO_2_ (Aerosil OX 50, Degussa) were mixed and H_3_BO_3_ was added as a flux. After grinding the mixtures in an agate mortar, they were fired in molybdenum crucibles for 3–5 h at ~1300°C in a furnace under a weak reductive atmosphere of flowing N_2_/H_2_ (5%) gas. After cooling down the synthesized samples to room temperature, they were ground again in an agate mortar. The final samples were irradiated with 365 nm UV-light for 5 min. After turning off the UV lamp, the emission spectra were recorded with a Hitachi 850 fluorescence spectrophotometer in the wavelength range from 300 to 950 nm.
